# Safety of bioabsorbable implants in vitro

**DOI:** 10.1186/s12893-015-0111-4

**Published:** 2015-12-12

**Authors:** Mehmet Isyar, Ibrahim Yilmaz, Gurdal Nusran, Olcay Guler, Sercan Yalcin, Mahir Mahirogullari

**Affiliations:** Department of Orthopaedic and Traumatology, Istanbul Medipol University School of Medicine, Bagcilar, 34214 Istanbul, Turkey; Department of Pharmacovigilance and Rational Drug Use Team, Pharmacologist Pharmacist, M.Sc. Republic of Turkey, Ministry of Health, State Hospital, 59100 Tekirdag, Turkey; Department of Orthopaedic and Traumatology, Canakkale Onsekizmart University School of Medicine, 17000 Canakkale, Turkey

**Keywords:** Bioabsorbable implant, osteocyte proliferation, osteoblastic activity, poly(lactic-co-glycolic acids)

## Abstract

**Background:**

The aim of the present study was to investigate the safety of bioabsorbable plates and screws in humans.

**Methods:**

For this purpose, an implant system based on [poly(lactic-co-glycolic acids)(85:15)] was designed. The system was tested for pH, temperature, and swelling and then its surface morphology was analyzed for surface porosity using environmental electron microscopy. Then, the effects of this bioabsorbable system on the viability and profileration of osteocytes were examined on a molecular level via in vitro experiments. A [poly(lactic-co-glycolic acids)(90:10)] bioabsorbable implant, which is commercially available and used in orthopedic surgery, was used as control group. For the statistical evaluation of the data obtained in the present study, the groups were compared by Tukey HSD test following ANOVA. The significance level was set as *p* < 0.05.

**Results:**

It was observed that the osteocytes cultivated on the PLGA system designed in the present study included more live cells and allowed more proliferation compared to the control.

**Conclusion:**

One of the criteria in the selection of implants for orthopedic surgery is that a good implant should not need removal and thus a second surgery. In the present study, a bioabsorbable implant was designed considering this criterion. The present study is the first step to prove the safety of this new design by in vitro toxicity and viability experiments.

## Background

Regarding the use of implants in orthopedic trauma surgeries, the followings are expected: stabilized fixation, minimal surface contact, and causing no foreign body reaction or toxicity. To avoid the extraction of an implant by a subsequent surgery for any reason, bioabsorbable implants have recently been gaining popularity [1–3].

The modern implants used in orthopedic surgeries help bone fixation morphologically, but the resorption on the surface has been one of the problems remaining unsolved [1, 2, 4].

Removal of an orthopedic implant requires surgery, which is not a desired process. The development of an innovative implant that can dissolve and be biologically absorbed after it is no longer needed has always been considered [2–5].

In the present study, bioabsorbable [poly(lactic-co-glycolic acids)(85:15)] (PLGA) composites were prototyped based on chitosan and imitating the combined plate and screw implant system. The biocompatibility of this implant was evaluated via an in vitro experimentation on osteocytes showing osteoblastic activity in order to determine whether it can be used in humans.

## Methods

This study was conducted with the permission of the Local Ethics Evaluation Commission of Medipol University, Medical Faculty. Signed informed consent forms were obtained from the participants to conduct research on tissues.

### Materials

Collagenase Type II enzyme (1 mg/mL; Invitrogen Corporation, USA), Hank’s Balanced Salt Solution 1X, 14025, Gibco (HBSS), RPMI–1640 (Sigma-Aldrich GmbH, Germany), DMEM (1000 mg glucose/L, Sigma Chemical St. Louis, USA) Penicillin-Streptomycin, fetal bovine serum (Sigma Chemical St. Louis, USA), Osteoblast Stimulator MesenCult-XF kit (MSC Basal Medium human-05431, Osteogenic Stimulatory Supplement human-05435, and β-Glycerophosphate human-05436), which is used as osteogenic progenitor, were obtained from Stemcell Technology, USA (≠9966/05434).

The laminar flow cabinet (Air Flow-NUVE/NF–800 R) and the incubator (NUVE) were obtained from Akyurt, Ankara, Turkey. Primary cell culture consumables (flask, 6-well plate, pipette, tube, and scraper) were obtained from Sarstedt, Greigner. A CKX41 invert microscope, and for ELISA measurements, a Mindray MR 96 A were used (PRC). A Quanta 250 FEG (Fei Company, Hillsboro, Oregon, USA) environmental scanning electron microscope (ESEM) was used. For porosity assessment of the plates and surface morphology characterizations, a FEI-Philips XL30 ESEM-FEG scanning electron microscope and gold-plated Polaron SC7640 Sputter Coater were used. The antibodies used for immune phenotyping were BD brand and they were analyzed using a BD FACS-Calibur flow cytometer.

### Experiment design

All experiments were performed three times. Primary osteocyte cultures, which were used for viability, profileration and toxicity experiments, were prepared fresh and replaced every other day.

**Fig. 1:**
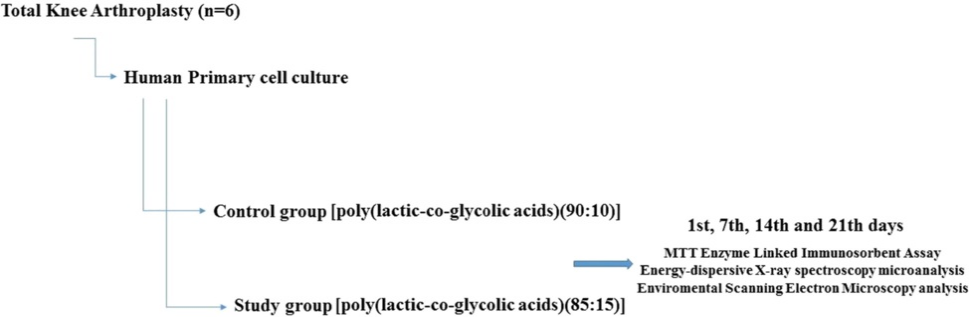
Experimental study design

The PLGA implant system was designed as prototype. Bioabsorbable [poly(lactic-co-glycolic acids)(90:10)] Lactasorb® implant, which is commercially available and can be used in orthopedic surgery, was used as control. Standard primary osteoblast cultures were obtained from the patients’ bone tissues (n = 6). Samples were prepared in sufficient numbers on days 1, 7, 14, and 21 for MTT-ELISA, energy-dispersive X-ray spectroscopy (EDS) microanalysis, SEM, and ESEM analyses (Fig. 1).

### Preparation of the PLGA-based plate and screw implant system and its placement in well-plates

Before the preparation of the prototype, that were imitating the PLGA plate and screw, polyethylene glycol 300 (PEG), hydroxyapatite (HA) and chitosan were dissolved in proper solvents. Then, using an assay balance, PLGA was prepared on a watch glass dish with a 85:15 lactide:glycolide ratio. PLGA beads placed in falcon tubes with %4 pure acetone (−CH3COO^−^) solution to have %6 concentration.

The solutions were vortexed three times for 1 min each during the 2-h waiting period, and then the solutions were taken into a porcelain beaker and kept in a hot water bath for 25 min keeping the temp below 50 °C. Ammonium thiosulfate was added to the solution (11 g/55 ml) made with chitosan in bovine serum and HCl (0.01 M). It was stirred using a glass rod. The hardness of the product was adjusted by the addition of poly(vinyl alcohol), chitosan, and ammonium thiosulfate, in this order, as cross linker. To obtain the hardness level that is suitable to be used in surgery, this process was repeated several times.

The samples to be used in well-plates were retrieved from the solvents using a vacuum desiccator at 38 °C. The samples were washed in sterile PBS and distilled water, and then dried by freezing. The samples were then sterilized in ethylene oxide.

### Analyses of the temperature, pH and swelling tests of the PLGA prototype designed

For the assessment of the swelling behaviors of the plates, the Flory Rehner theory was used [6, 7]. For this purpose, samples were cut into pieces with a 9-mm diameter and 2-mm height. These samples were put in beakers with phosphate buffer (at 37 °C, pH, 7.4). The samples were dried using blotting paper several times in the 21-day waiting period and they were weighted. Hydration rates were calculated using the data obtained in this period [8].

To obtain the swelling curves of the plates, the data obtained gravimetrically by the Swelling rate = (Wt-W_0_)/W_0_ equation were plotted against time (W_0_ = Dry weight of the sample, Wt = Wet weight of the sample at time t).

The plates were placed in hot water baths at 25 °C and 50 °C and the diameter changes were measured by an electronic calipers and the data were plotted. The diameter changes in PBS solutions with different pH values (6, 6.5, 7, 7.4, 7.5, 8) at 37 °C were measured and recorded [9–11].

### Obtaining the primary osteoblasts

**Fig. 2:**
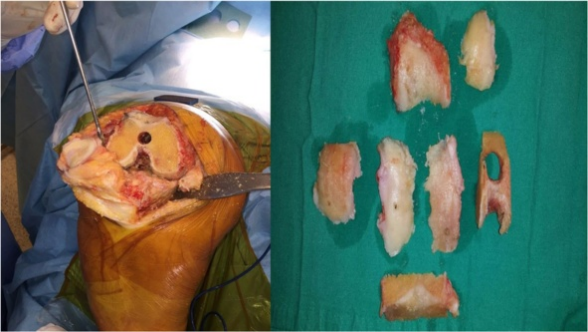
Osteochondral tissue obtained during total knee replacement surgery

The patients (n = 6, mean age = 64) included in the present study were diagnosed with severe osteoarthritis based on Kellgren and Lawrence Scale [12] and who did not respond to medical and conservative treatment protocols. Bone tissues obtained from these patients who underwent total knee arthroplasty were used to prepare the primary osteocyte cultures (Fig. 2).

These tissue parts were transferred in DMEM containing %5 penicillin. They were transferred to the laboratory at 4 °C in proper conditions. The bone tissue cells taken into the flow cabinet were irrigated in PBS solution and red blood cells were separated by minisart filters. The bone tissues divided into 0.3 cm^3^ pieces were washed with 0.1 % (a/h) HBSS solution. The samples were taken into tubes per the manufacturer’s instructions and 400 μg Clostridium Histolyticum-based collagenase type II enzyme dissolved in HBSS solution was added. The samples were kept in an incubator with 5 % CO2 and at 37.4 °C for a day and they were centrifuged at 4 °C and 1300 rpm for 15 min. The cell pellets deposited at the bottom were resuspended using the culture medium prepared with RPMI-1640. They were left for incubation after transferred to flasks.

Until the third passage, the medium of the cells were replaced every other day with the following culture content: FBS, Penicillin-Streptomycin, and RPMI-1640.

Osteogenic stimulator kit was used for the osteoblastic activity to occur. Following the second passage, for 16 days, the medium of the cells were replaced every other 4 days with the following culture content: 47.5 ml MSC Basal Medium, 2.5 ml Osteogenic Stimulator Supplement, and 1 Molar 175 microliter β-Glycerophosphate, FBS, Penicillin - Streptomycin, DMEM.

The cells attaining to minimum 90 % confluency, adhering to the bottom, and with completed immunoflow cytometric analyses were taken for experimentation. Then, the samples were placed in well pates (65X10^3^ cells/well) by counting on Thoma lams with trypan blue.

### Invert light microscopy images

Microphotographs of the cell organizations were taken at 4X, 10X, 20X and 40X zoom levels before they were placed into cell plates using an invert light microscope. The images were assessed using Olympus cell softimaging System software.

### ESEM and SEM analyses

Culture medium in the well plates were discarded. For the fixation of the cells, 2.5 % glutaraldehyde solution containing 97.5 ml cacodylate buffer and 2.5 ml glutaraldehyde was added to the wells to cover the samples. The glutaraldehyde solution was removed off the samples with a pipettor gun after 2 hours of waiting period at room temperature. The samples were washed with cacodylate buffer 3 times. The samples were covered again with cacodylate buffer after the last wash and kept at 4 °C in a refrigerator until measurement.

The cells in the control group were directly evaluated with ESEM microscope, and the cells placed on plates were coated with gold and evaluated with SEM. The samples were cooled down to −20 °C to perform these analyses. They were dried by holding the samples overnight in a Freeze-dryer, [6, 13–16]. Then, the samples were coated with 5 nm gold. Images were taken at various zoom levels while working at 10 kV.

### Energy-dispersive X-ray spectroscopy (EDS) microanalysis

In the analysis method that is used for elemental analysis or chemical identification, the surface of the sample is bombarded with high-energy electrons. Following this collision, due to the electron discharge, the movement of electrons from the outer orbitals to the inner ones to fill in gaps is activated to make the chemical structure stable again. In the meantime, the chemical sample that lost energy reflects this energy loss by emitting X-ray. The energy and wavelength of this X-ray is not only related with the atom but also a characteristic feature of the orbitals with which the atom is exchanging electrons [15, 16].

X-rays originated from the chemical samples are detected by the semi-conductor detector. The electrons passed onto the conductivity band convert to electricity signals.

By only selecting the X-ray peaks of the respective element and by counting the X-rays via the EDS detector, for each point on the sample surface, the relative ratio of the element on the surface of the sample can be determined. The 2-dimensional map of these measurements show the X-ray map of the element of the chemical [15, 16].

To this end, chemical stability of the implants was assessed by carbon, hydrogen, and nitrogen percentages in the compounds of the control group and the PLGA prototype. By this way, whether the new implant system hosts the live cells as well as the commercially available counterparts was also evaluated.

### Immunophenotyping by flow cytometry

Before passaging (phase 4), the general condition of the cells cultured in DMEM medium with dexamethasone (100 nM), 05 μM ascorbate-2-phosphate, and 10 mM beta glycerophosphate was assessed by invert microscopy and reported. The osteoblasts adhering to the surface were scraped with 0.25 % Trypsin - EDTA and a scraper, and transferred to tubes. They were centrifuged twice at 4 °C and 2000 rpm for 5 minute each. Supernatants in the 15-cc falcon tubes were discarded. The pellets on the bottom were resuspended with the cell culture medium content. The cells were counted and 0.5X10^5^ cells were incubated in each well (at 22.4 °C, in a dark environment, for 20 min) with fluorescein isothiocyanate (FITC)-phycoerythrin (PE) that is specific to surface markers, conjugated monoclonal antibodies (HLA-DR, CD34, CD45, CD117 and CD44), and proper isotype controls.

At the end of the time period, the medium content was cleared from artifacts by centrifuging at 4 °C and 1300 rpm with the addition of pH = 7.4 PBS including 0.1 % sodium azide. The resuspended cell suspensions were evaluated using the flow cytometry device. The data were analyzed with BD Cell Quest Software (Safe Net Sentinel-CQPro-SRB) and the images were reported.

### Determination of viability and toxicity effects: MTT-ELISA analyses

Viability tests were carried out using the commercially available MTT kit per the manufacturer’s instructions. The working principle of the test is that tetrazolium rings in live cells are crashed by the dehydrogenase enzymes found in the mitochondria, which forms blue-colored formazan crystals, and it does not occur in dead cells. One extra well from each cell group and the control was set aside for this analysis. In a dark environment, on days 1, 7, 14, and 21, the medium in each of the well was discarded using a pipette gun. In each well, 500 μl culture medium solution containing MTT (3-[4,5- dimethylthiazol −2-yl]-2,5-diphenyltetrazolium bromide) and DMEM (1:6) was placed and they were incubated for 3 days. At the end of this time period, to end the reaction, 25 μl 10 % SDS was added into each well.

200 μl MTT solutions were extracted and transferred to 96-well plates. Then the samples were analyzed using the ELISA device at 490 nm and the results were reported.

This test was used to check whether the cells survive and maintain healthy proliferation in the PLGA prototype compared to the control group.

The proliferation of the cell cultures grown in the designed implant was calculated as percentage (%) using the following equation:

Proliferation was assumed to be 100 % in the control group on day 1, which was taken as baseline. Live cell numbers were also reported for days 1, 7, 14, and 21 as percentage (%) and the differences between the PLGA prototype and the control group were investigated.

### Statistical analysis

In the analysis of the data, results were considered in terms of cell proliferation. The results were expressed as mean ± standard deviation. Minitab 16 was used for statistical analysis. One-way ANOVA test was used for inter-group comparisons. To test the significance of any meaningful relationship, Tukey HSD test was used. Alpha significance level was set as <0.05.

## Results

### An evaluation of the temperature, pH, and swelling test results of the PLGA prototypes

**Fig. 3:**
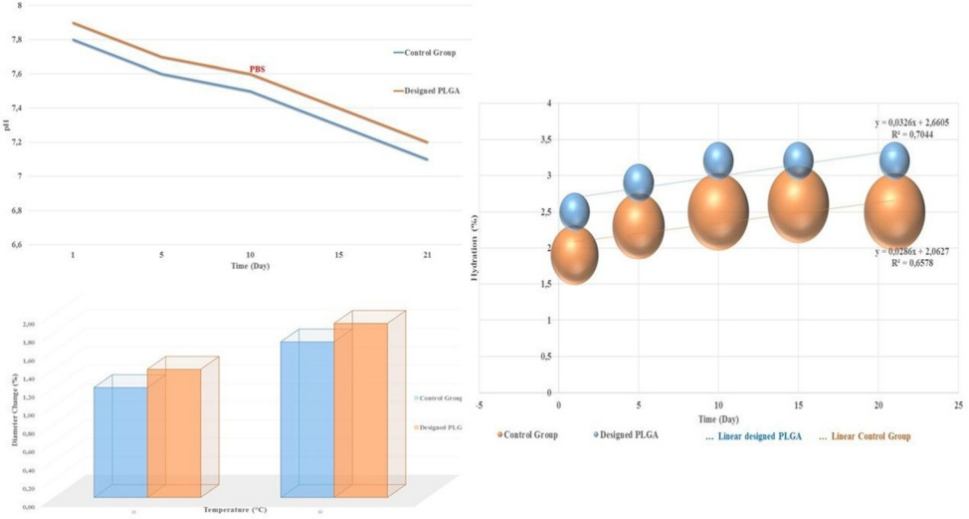
Evaluation of the temperature, pH, and swelling test results of novel designed PLGA composites

Temperature and pH analyses showed the degradation of the PLGA implant in PBS and different pH levels (Fig. 3).

pH change indicates that the PLGA-based plate samples have a high buffering capacity.

### Invert light microscopy evaluation

**Fig. 4:**
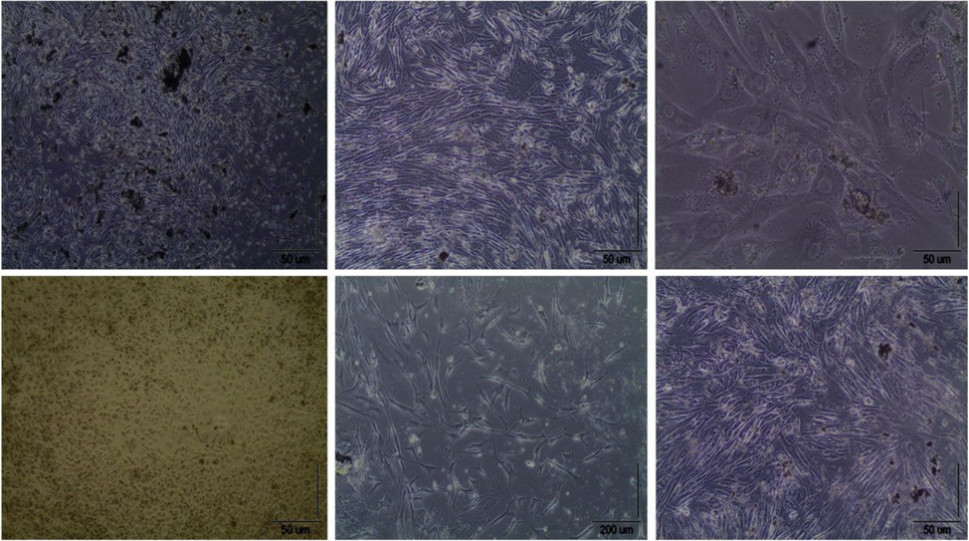
Microphotographs of the cell organizations were taken at 4X, 10X, 20X and 40X zoom levels before they were placed into cell plates using an invert light microscope

Before the subculturing that is required for immunophenotyping, invert microscope images of the osteocytes showed that they were healthy in 90 %;15/16 confluent ratio (Fig. 4).

### Evaluation of the surface morphologies (with and without cells) of the designed PLGA by SEM and the cells by ESEM

**Fig. 5:**
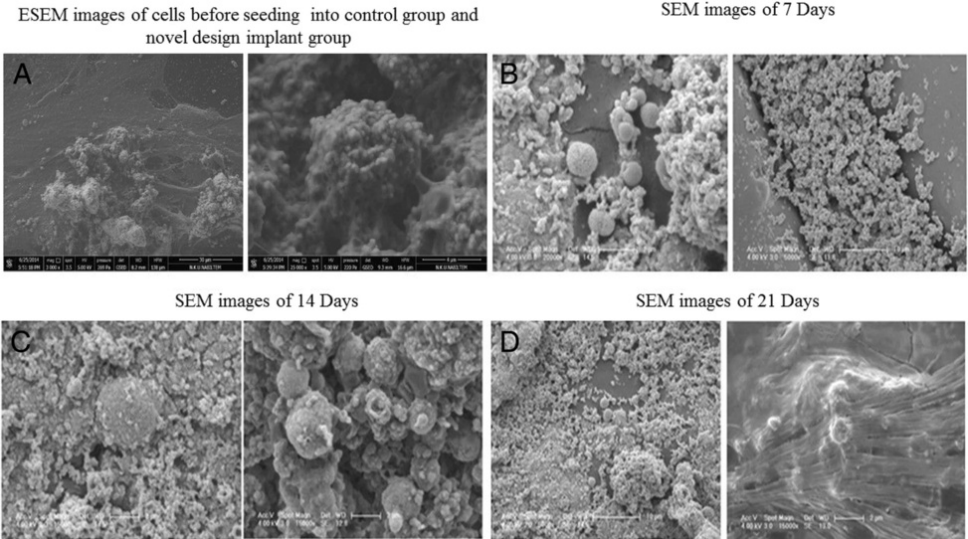
Morphological evaluation of osteocytes on the control and novel designed PLGA groups. **a**: ESEM images of cells before seeding into cntrol group and novel design implant group. **b**: SEM images of 7 days. **c**: SEM images of 14 days. **d**: SEM images of 21 days

The morphological evaluation of the surface and cross-sectional structure of the plates and the SEM analysis (to inquire about the cellular nesting) showed large pores inside the plates (Fig. 5).

In the PLGA prototype, on figure the number and distribution of cells in the cracks that occurred after the dry off were regular and homogeneous compared to the control group. Besides, the density and extension of the extracellular matrix covering the PLGA were noteworthy on day 21.

**Fig. 6:**
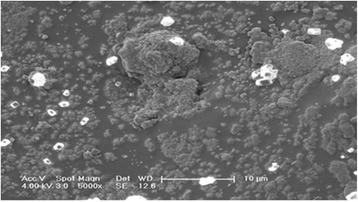
Energy-dispersive X-ray spectroscopy (EDS) microanalysis Quantification analysis at 5000X

EDS test results, which was performed after the scanning electron microscopy, showed dense, cubical, shiny calcium zones (Fig. 6).

EDS analysis of the samples with no cells showed that the PLGA implant system contained more carbon atoms (an indication of chemical stability) and more oxygen atoms allowing more oxygen transmission (an indication of a better growth medium for cells) compared to the control group (Table 1).

**Table 1:** Energy-dispersive X-ray spectroscopy (EDS) microanalysis Quantification

Element (Wt %)	Controlgroupwithcells	A noveldesignPLGA groupwithcells
C K	17.64	24.87
N K	13.11	19.70
O K	26.74	26.67
Na K	10.13	6.54
Mg K	16.13	4.63
P K	6.56	5.65
Ca K	9.69	11.94
TOTAL	100	100

C: Carbon, N: Azote, O: Oxygen, Na: Sodium, Mg: Magnesium, P: Phosphorus, Ca: Calcium are the chemical symbol.

EDS analysis of samples which include cellss how ed that, PLGA prototype is a good host. Azote atom rate, which is the indicator of cell viability, was 19.70 % in the PLGA group. Novel design PLGA permitted beter osteoblast proliferation.

### Assessment of the immunophenotyping by flow cytometry

In addition to MTS ELISA analyses, the osteoblastic activity specific to bones was proven by proliferation and differentiation flow cytometry analysis by the help of fluorescein isothiocyanate (FITC) - phycoerythrin (PE), that is specific to cell surface markers, conjugated monoclonal antibodies ( HLA-DR, CD34, CD45, CD117 and CD44), and proper isotype controls. It was observed that MTS-ELISA results were in line with flow cytometry results (Table 2).

**Table 2:** Expression rates of various antigens that obtained from flow cytometry by immunophenotyping and avarage fluorescent density at primary cell culture

Antigens	Case 1	Case 2	Case 3	Case 4	Case 5	Case 6	Exp	M ± SD
HLA-DR	0	0	0	0	0	0	(−)	0
CD34	0	0	0	0	0	0	(−)	0
CD117	0	0	0	0	0	0	(−)	0
CD45	0	0	0	0	0	0	(−)	0
CD44	98.71	97.93	96.29	95.69	98.89	99.71	(+)	%97.87 ± 1.57

(M: Mean & SD: Standard deviation. Exp: Expression)

Following the third phase subculture of the cells isolated from human primary osteocyte and osteoblasts, HLA-DR, CD34, CD117 and CD45 markers showed negative expression and CD 44 marker showed positive expression.

### Statistical evaluation of viability, proliferation, and toxicity by MTT-ELISA

**Fig. 7:**
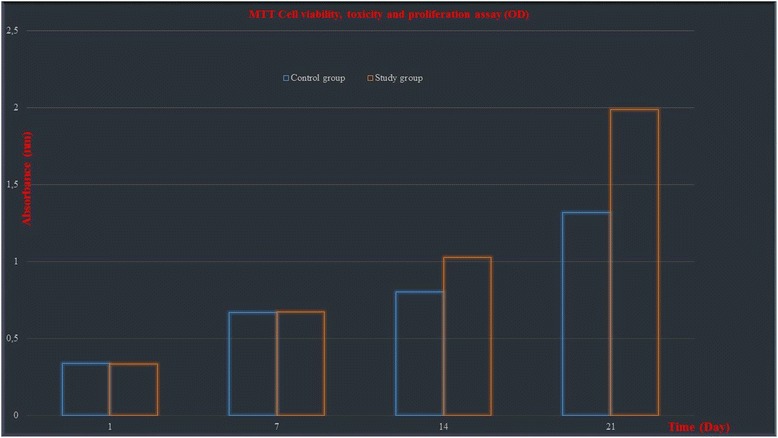
Bone cell viability (MTT assay; 540-nm absorbance) at 1., 7., 14., and 27. Days

In the evaluations regarding the cell proliferation, as the cell viability was reported on days 1, 7, 14 and 21 as percentage (%), which group showed the best osteoblastic activity and growth was reported (Fig. 7).

## Discussion

Recently, application of the polymeric biological systems gained increased popularity [17]. Current literature showed that, metal implant systems are considered as the first choice in the treatment protocols due to their mechanical strength but they may exert toxic effects on the viable cells in the host by inflammation and deposition of metabolites secondary to the hydrolysis of the metallic materials [18].

The choice of the type of material that should be used in trauma patients recently became more important due to the possible positive and negative impact of the material on both the well being of the host and the bone remodelling. Biomaterals seems to be advantageous for the patients because they do not necessitate another operation for extraction and they may permit bone formation in the site of elution.

Biomaterial design history in the literature shows that the use of some materials, such as calcium phosphate, was investigated because it was hypothesized that they may simulate theosteogenesis, but it was observed that they do not induce the bone development but they are suitable for hard tissue formation [19]. Tricalcium phosphate-beta-based implant systems were used, no regeneration was reported in trials and they formed connective tissue capsules [20, 21]. In addition, hydroxyapatite (HA)- coated screws were reported to be used successfully [22].

Therefore, in the preparation of the PLGA-based implant system in the present study, HA was used to make the composite instead of tricalcium phosphate-beta. To increase the porous features of the tissue scaffold made with hydrophobic polymers, it was reported that water is added to the polymer solutions [23, 24].

In the present study, for the preparation of the polymer prototype, after freezing at −80 ˚C for 48 hours, the material was dried. The water added to the solution later was removed to obtain a more stable surface area.

In the literature, in the design of such biomaterial systems, osteoinductive and osteoconductive materials, such as HA and chitosan, have been used. By this way, bone and capillary network formation is allowed yielding a 3-dimensional structure boosting cell proliferation, which has been thought to be a proper option for the treatment of bone fractures [25–28].

Therefore, in the present study, in addition to PLGA, which has osteoconductive featuresprotecting the osteoblasts or the cells that my convert to osteoblasts, HA and chitosan were usedin the making of the implant prototype.Before the use of any new design in humans, individual chemicals involved in the design or theentire compound is assessed in culture medium, and partial atypical cell development may causechanges in the cell culture content, which may result in cell loss [29].

As it is well known, PEG's are frequently used in a number of medical procedures (designation of surface active materials and etc.) to provide polymerization and preferred due to their high biocompatibility [30, 31].

However, we did not use PEG 300 ( a PEG derivative ) in an attempt to design a surface active agent - as you indicated. We used is as an adjunct binding materials to promote polymerization of the designed PLGA and consolidate the weak crosslinking that occured with chitosan (provision of increased binding by increasing the hydrophilic regions). PEG 300 used in our design is 1/50 mole/mole and it is usually neglected hence it is in trace amounts.

Even though the fact that in vitro experimental analyses in the present study were performed in culture medium may be considered as a limitation of the study, the cells used here are primary cell cultures obtained from human bone tissues, not from cell-line or animal tissues, whichmakes the data more robust. The actual limitation of the study was the lack of biomechanical tests of the implant. However, in this first pilot study, in vitro toxicity and viability tests were aimed, and subsequent studies were planned to be carried out regarding the animal experimentsand experiments to present the biomechanical features.

Debates exist regarding the mechanical stability and complication rates of the biocompatible materials but in a recent study Bos and Smart et al. fixated the mandibular fractures with biocompatible materials and they favoured their use due to their strenth and contributions to bone remodelling [32, 33].

Design of novel orthopaedic bioimplants that resides in body fluids and testing their biocompatibility have a paramount importance in modern medicine. Biomaterials that lack the desired biological properties may not be accepted as successful even if they carry the appropriate physico-chemical properties

When polymeric implants are utilized, tissue – polymer interaction should be taken into account. A number of in vivo and in vitro studies dealing with these interacitons were performed [34, 35].

In these researches, many molecules including but limited to PEG-poly(ε-caprolactone) [36], HA, chitosan, were used to avoid cellular toxicity in the host [36–40]. For these reasons, in an in vitro setting, we aimed to investigate the possible detrimental effects of the ingredients of the bioimplant that could be observed after application.

The ingredients of the designed bioimplant should have certain properties. Physical, chemical and thermal properties of the designed biomaterial such as the expected weight of the implant after application due to swelling ( % hydration) and its attitude against the pH changes in acidic, basic or neutral milleu should be well tested in vitro before proceeding to animal or human studies [41].

After these studies were conducted and once the components were proved to have no cytotoxic potential, then studies investigating its physico-chemical properties such as the extent of change in the loss of elasticity and friability.

The buffering capacity of the designed implant was found to be superior to the controls in our study which was represented by the changes measured in pH. This difference was statistically significant (p < 0.05).

Bernstein et al. incubated bioresorbable implants containing poly-L-lactide-co-glycolic acid/β-tricalciumphosphate (PLGA/TCP) (70 %/30 %) with human osteoblast-like cells. They observed no associated cytotoxicity and witnessed more contact points under ESEM in comparison to controls [35].

Bernstein et al., adhesion and proliferation of human osteoblast-like cells on different biodegradable implant materials used for graft fixation in ACL-reconstruction.

We used PLGA (85/15) in our experiment and our results were superior to control (PLGA with a ratio of 90/10) in terms of cell viability and proliferation in MTT ELISA and ESEM analysis.

Meyer et al. pointed out that the use of degredable composite materails in orthopaedics is a significant issue and they evaluated the effcts of PLGA degradation products on human osteoblasts in vitro. They concluded that the toxicity of the polymer degradation products was limited to only high concentrations [42].

In our study, in concordance with the existing literature, the ingredients of the designed implant was not cytotoxic.

As a result, in the present study it was observed that the PLGA-based prototype system biodegraded and the samples showed a high buffering capacity. The design was observed to possess the porosity and surface allowing the cells to nest. Dense and shiny calcium zones showed that the PLGA prototype contained more carbon atoms compared to the control group, which was an indication of chemical stability. Moreover, it was noteworthy to observe that the material also contained more oxygen, which would boost the cell growth. One of most important thing that, , the PLGA prototype has more nitrogen atom numbers, which was the proof of viability. CD44, which is the symbol for osteopontin and hyaluronan and also the indication of osteoblastic activity, showed high and positive expression. The tests showed that the new design is not toxic in vitro.

## Conclusion

One of the criteria in the selection of implants for orthopedic surgery is that a good implant should not need removal and thus a second surgery. In the present study, a bioabsorbable implant was designed considering this criterion. The present study is the first step to prove the safety of this new design by in vitro toxicity and viability experiments.
